# Biosynthesis of CdS Quantum Dots Mediated by Volatile Sulfur Compounds Released by Antarctic *Pseudomonas fragi*

**DOI:** 10.3389/fmicb.2019.01866

**Published:** 2019-08-13

**Authors:** Carla Gallardo-Benavente, Ornella Carrión, Jonathan D. Todd, Joana C. Pieretti, Amedea B. Seabra, Nelson Durán, Olga Rubilar, José M. Pérez-Donoso, Andrés Quiroz

**Affiliations:** ^1^Programa de Doctorado en Ciencias de Recursos Naturales, Universidad de La Frontera, Temuco, Chile; ^2^Centro de Excelencia en Investigación Biotecnológica Aplicada al Medio Ambiente (CIBAMA), Facultad de Ingeniería y Ciencias, Universidad de La Frontera, Temuco, Chile; ^3^School of Environmental Sciences, University of East Anglia, Norwich, United Kingdom; ^4^School of Biological Sciences, University of East Anglia, Norwich, United Kingdom; ^5^Centro de Ciencias Naturais e Humanas, Universidade Federal do ABC, Santo André, Brazil; ^6^Institute of Biology, Universidade Estadual de Campinas, Campinas, Brazil; ^7^Departamento de Ingeniería Química, Universidad de La Frontera, Temuco, Chile; ^8^BioNanotechnology and Microbiology Lab, Center for Bioinformatics and Integrative Biology, Facultad de Ciencias de la Vida, Universidad Andres Bello, Santiago, Chile; ^9^Departamento de Ciencias Químicas y Recursos Naturales, Facultad de Ingeniería y Ciencias, Universidad de La Frontera, Temuco, Chile

**Keywords:** Antarctic bacteria, quantum dot, nanoparticle biosynthesis, volatile sulfur compounds, cadmium sulfide

## Abstract

Previously we reported the biosynthesis of intracellular cadmium sulfide quantum dots (CdS QDs) at low temperatures by the Antarctic strain *Pseudomonas fragi* GC01. Here we studied the role of volatile sulfur compounds (VSCs) in the biosynthesis of CdS QDs by *P. fragi* GC01. The biosynthesis of nanoparticles was evaluated in the presence of sulfate, sulfite, thiosulfate, sulfide, cysteine and methionine as sole sulfur sources. Intracellular biosynthesis occurred with all sulfur sources tested. However, extracellular biosynthesis was observed only in cultures amended with cysteine (Cys) and methionine (Met). Extracellular nanoparticles were characterized by dynamic light scattering, absorption and emission spectra, energy dispersive X-ray, atomic force microscopy, transmission electron microscopy, X-ray diffraction and X-ray photoelectron spectroscopy. Purified QDs correspond to cubic nanocrystals of CdS with sizes between 2 and 16 nm. The analysis of VSCs revealed that *P. fragi* GC01 produced hydrogen sulfide (H_2_S), methanethiol (MeSH) and dimethyl sulfide (DMS) in the presence of sulfate, Met or Cys. Dimethyl disulfide (DMDS) was only detected in the presence of Met. Interestingly, MeSH was the main VSC produced in this condition. In addition, MeSH was the only VSC for which the concentration decreased in the presence of cadmium (Cd) of all the sulfur sources tested, suggesting that this gas interacts with Cd to form nanoparticles. The role of MeSH and DMS on Cds QDs biosynthesis was evaluated in two mutants of the Antarctic strain *Pseudomonas deceptionensis* M1^T^: *megL*^–^ (unable to produce MeSH from Met) and *mddA*^–^ (unable to generate DMS from MeSH). No biosynthesis of QDs was observed in the *megL*^–^ strain, confirming the importance of MeSH in QD biosynthesis. In addition, the production of QDs in the *mddA*^–^ strain was not affected, indicating that DMS is not a substrate for the biosynthesis of nanoparticles. Here, we confirm a link between MeSH production and CdS QDs biosynthesis when Met is used as sole sulfur source. This work represents the first report that directly associates the production of MeSH with the bacterial synthesis of QDs, thus revealing the importance of different VSCs in the biological generation of metal sulfide nanostructures.

## Introduction

Nanotechnology is the study, understanding, control and restructuring of matter on the scale of nanometers (size range 1–100 nm) to create materials with new properties and functions that remarkably differ from their bulk counterpart (Sanchez and Sobolev, 2010). Nanoscience research and its applications, developed in recent decades, have significantly affected a number of areas, such as information technology, agriculture, energy, environmental science, medicine and food safety, among others (Solomon, 2018). Consequently, there is a growing need to develop nanoparticles by using new, cheaper, reliable and eco-friendly synthesis methods that do not involve toxic chemicals. To achieve this, research on the use of natural sources such as biological systems becomes essential. Biological production or biosynthesis of metal nanoparticles has been widely studied through the use of microorganisms, such as fungi, yeast and bacteria (Wu et al., 2015; Chakraborty et al., 2018; Qin et al., 2018). Due to the convenience of this method, a cost-effective, environmentally friendly and highly biocompatible alternative based on the use of bacterial cells has emerged (Monrás et al., 2012).

Bacteria of the *Pseudomonas* genus have been employed as cell factories to produce different types of metal nanoparticles. In fact, several *Pseudomonas* strains have been reported to produce extracellular gold (Au) and silver (Ag) nanoparticles (Kumar and Mamidyala, 2011; Baker et al., 2015; Ali et al., 2016; Jo et al., 2016). In addition, some *Pseudomonas* species can also synthesize a specific type of nanoparticles termed quantum dots (QDs), constituted of cadmium selenide (CdSe) (Ayano et al., 2015) or cadmium sulfide (CdS) (Gallardo et al., 2014; Plaza et al., 2016; Oliva-Arancibia et al., 2017).

Quantum dots or semiconductor nanocrystals are bimetallic structures that generally contain II–VI or III–V elements such as CdS, CdSe, ZnS, ZnTe, CdTe, InP or GaAs (Mal et al., 2016; Jadhav et al., 2017; McHugh et al., 2018). The size and composition of the nanoparticles are responsible for their unique physical, chemical and optical properties, which arise through quantum confinement effect (Alivisatos, 1996; Rengers et al., 2019). The remarkable properties of QDs, such as broad absorption, narrow and size-dependent emission spectra, resistance to photobleaching, strong luminescence and long luminescent lifetimes (Alivisatos, 1996; Zhou and Ghosh, 2007) allow their use in a number of technology-based applications of high economic, technological and biological value as for example optoelectronics (Faraon et al., 2007), solar cells (Nozik et al., 2010; Muthalif et al., 2019), imaging techniques (Wagner et al., 2019) and quantification of different molecules (Durán-Toro et al., 2014; Nguyen et al., 2015), among others.

Bacterial biosynthesis of cadmium-based QDs has been extensively studied in the past few years through the biological production of both intra- and extracellular nanoparticles (Plaza et al., 2016; Ulloa et al., 2016; Yang et al., 2016; Oliva-Arancibia et al., 2017), as well as to study the development of other nanoparticles and the mechanisms associated with biosynthesis (Venegas et al., 2017). However, the mechanism involved in Cd-based QD biosynthesis is still unknown. Overall, to carry out the biosynthesis of CdS nanoparticles, cadmium ion (Cd^2+^) should be added into the system to react with sulfide anion (S^2–^) provided exogenously or by the bacterial metabolism, in order to form the nanocrystal (Bai et al., 2009a; Gallardo et al., 2014; Yang et al., 2016).

Some bacteria are capable of releasing hydrogen sulfide (H_2_S) as a strategy to trap exogenous cadmium (Cd) to form less toxic insoluble metal-sulfides (Holmes et al., 1997). This ability has been widely used in the biosynthesis of QDs during the last years (Bai et al., 2009b). In general, the biosynthesis of CdS QDs has been associated with sulfur-containing molecules such as glutathione, peptides, Cys and H_2_S (Monrás et al., 2012; Yang et al., 2016). However, the role of other volatile sulfur compounds (VSCs) produced from bacterial metabolism in QDs biosynthesis, such as dimethyl sulfide (DMS), dimethyl disulfide (DMDS) and methanethiol (MeSH) (Schulz and Dickschat, 2007), has not been studied yet.

Recently, intracellular biosynthesis of CdS QDs has been reported at low temperatures (15°C) using Antarctic bacteria from the *Pseudomonas* genus. Nanoparticle synthesis was performed in the presence of CdCl_2_ without any additional sulfur sources. The mechanism of CdS formation was attributed to the production of sulfide from H_2_S by Cys desulfhydrase in all strains tested except for *Pseudomonas fragi* GC01 strain (Gallardo et al., 2014). In this work, we study the link between the production of VSCs and the biosynthesis of CdS nanoparticles in *Pseudomonas fragi* GC01. We also carry out for the first time the biosynthesis of QDs in bacteria using Cys and Met as sole sulfur sources.

## Materials and Methods

### Bacterial Strains and Culturing

Strains used in this work were *P. fragi* GC01 (Gallardo et al., 2014), *Pseudomonas deceptionensis* M1^T^ wild type, *P. deceptionensis* M1^T^ *megL*^–^ and *P. deceptionensis* M1^T^ *mddA*^–^ (Carrión et al., 2011, 2015). Bacterial strains were grown in Luria-Bertani (LB; complete) medium (Sambrook and Russell, 2001) for 24 h or in M9 (minimal) medium (Sambrook and Russell, 2001) for 48 h (stationary phase culture) at 28°C. *P. deceptionensis* M1^T^ *megL* and *mddA* mutants were grown in media containing kanamycin (20 μg mL^–1^) and spectinomycin (800 μg ml^–1^), or gentamicin (5 μg mL^–1^), respectively.

### Evaluation of CdS QDs Biosynthesis

Nanoparticle biosynthesis by bacteria was performed following the protocol described by Monrás et al. (2012) and Gallardo et al. (2014). Briefly, bacteria were grown in M9 minimal medium supplemented with 0.25 mM of six different sulfur sources [sulfate (MgSO_4_), sulfite (Na_2_SO_3_), thiosulfate (Na_2_O_3_S_2_), sulfide (Na_2_S), Met and Cys] for 48 h. Then, cultures were centrifuged at 10,000 rpm for 5 min (Himac CT15E centrifuge). Bacterial pellets were resuspended in CdCl_2_ (10 μg mL^–1^) and incubated at 28°C for another 48 h before assaying the production of nanoparticles. Nanoparticle formation was assessed using a short-wave UV-transilluminator at λ_excitation_ = 360 nm. Analyses were done with three biological replicates, and samples with no CdCl_2_ added were included as a control.

### Sulfide Detection Assay

The production of H_2_S was evaluated using lead acetate-soaked papers, as described by Shatalin et al. (2011). The assay was performed using 1 mL of bacterial cultures in microcentrifuge tubes and a lead acetate paper attached under the cap. Briefly, bacterial strains were grown on M9 minimal medium at 28°C until reaching an OD_600_∼0.8. Then, cultures were washed with fresh M9 medium with no sulfur sources. Bacterial pellets were resuspended and inoculated into M9 medium containing sulfate, sulfite, thiosulfate, sulfide, Met and Cys (0.5 mM) as sole sulfur sources in the presence or absence of CdCl_2_ (10 μg mL^–1^). Tubes were covered with a paper embedded in lead acetate (100 mM) and incubated at 28°C for 2 h before detecting sulfides as described in Gallardo et al. (2014). Controls consisted of samples incubated with no sulfur sources.

### Evaluation of Extracellular Biosynthesis of CdS QDs

The study of extracellular biosynthesis of nanoparticles was carried out following the protocol described by Monrás et al. (2012) and Gallardo et al. (2014) with some modifications. Briefly, cells were grown for 48 h in M9 medium at 28°C. Then, cultures were centrifuged at 10,000 rpm for 5 min (Himac CT15E centrifuge) and bacterial pellets were resuspended in fresh M9 medium supplemented with CdCl_2_ (20 μg mL^–1^) and a sulfur source such as Cys (0.1–2 mM), SO_4_^2–^, SO_3_^2–^, S_2_O_3_^2–^ or Met (0.25–50 mM). After 0.5–2 h of incubation with Cys and 72 h with the other sulfur sources at 28°C, cells were centrifuged for 5 min at 10,000 rpm. Fluorescence of the supernatant was measured using a short-wave UV-transilluminator at λ_excitation_ = 360 nm. Cultures of bacterial strains were set up in triplicate and samples with no CdCl_2_ were used as controls.

### Purification of Biosynthesized CdS Nanoparticles

*Pseudomonas fragi* GC01 was grown in M9 medium at 28°C in the presence of Cys (2 mM) or Met (40 mM) as sole sulfur sources to study the production of extracellular QDs. A total of 10 mL of culture was taken after 1, 2 and 3 h incubation with Cys and after 72 h incubation with Met. Samples were then centrifuged 15 min at 6,000 rpm (PrO-Research K241R centrifuge) before collecting the fluorescent supernatants. Supernatants were filtered through 0.2 μm filters (BioLab) to remove cellular debris. Filtered supernatant was then concentrated to 100 μL using a 10 KDa membrane (Amicon). Concentrated nanoparticle suspension was lyophilized overnight and stored at 4°C until use.

### Characterization of CdS Nanoparticles

#### Absorption and Fluorescence Spectroscopy

Absorbance and fluorescence spectra of purified nanoparticles were determined with a multiplate reader Synergy H1 M (BioTek). Measurements were performed at room temperature and absorbance spectra were recorded in the range of 300 to 700 nm, whereas emission spectra were obtained using λ_excitation_ = 360 nm and recorded in the range of 390 to 700 nm (Gallardo et al., 2014; Plaza et al., 2016).

#### Quantum Yield (QY) Determination

The quantum yield (QY) of purified CdS QDs was determined following the protocol described by Venegas et al. (2017) and Bruna et al. (2019). Briefly, the fluorescence of two samples of QDs with different absorbances (between 0.01 and 0.1) was determined after excitation at 360 nm. The procedure was applied for yellow and red nanoparticles (obtained from Cys and Met, respectively) dissolved in water and as reference fluorescein dissolved in ethanol (QY = 0.97). Emission spectra were measured to obtain the integrated fluorescence intensity (IFI, defined as the area of the fluorescence spectrum), and this value was plotted versus the absorbance of the solution. The slope of both curves (*m*) and the refractive index of the solvents (*n*) (water: 1.333 and ethanol: 1.335) were used to calculate the QY of CdS QDs considering fluorescein as reference (R). The following equation was used:

QY=NPsQY[m/NPsm]RR[n/2NPsn]2R

#### Dynamic Light Scattering (DLS) Measurements

The hydrodynamic size, zeta potential and polydispersity index (PDI, which is an indicative of size heterogeneity) were evaluated by dynamic light scattering (DLS) using the Zetasizer Nano ZS (Malvern Instruments Co., United Kingdom). Polystyrene cuvettes with a path length of 10 mm at 25°C and a refraction index of 2.6 were used.

#### Atomic Force Microscopy (AFM)

Atomic force microscopy (AFM) images of nanoparticles were obtained using an AFM/SPM Series 5500 dynamics microscope (Agilent Technologies). Nanoparticles were measured using a non-contact mode probe of 4 nm thickness, 125 μm in length, 30 μm frequency of 320 kHz resonance and a force constant of 42 N/m. Samples were 1/100 diluted and drop-casted over a silicon substrate before determining the average size of the nanoparticles at solid state using the WSxM 5.0 software, by automatically counting an average number of 40 particles per image for each CdS sample (Horcas et al., 2007).

#### Transmission Electron Microscopy (TEM)

The TEM analysis of nanoparticles was performed at the Center for the Development of Nanoscience and Nanotechnology (CEDENNA), using a HITACHI HT 7700 transmission electron microscope, operating with a Tungsten filament at an accelerating voltage of 120 kV, allowing a resolution of 0.2 nm. Samples (powder) were dispersed in distilled water using an ultrasonic bath and deposited on a copper TEM grid (200 mesh) coated with Formvar/carbon. Water was subsequently evaporated at room temperature.

#### Energy Dispersive X-Ray (EDX)

The elemental composition of the synthesized nanoparticles was determined with a scanning electron microscope (JSM-6010LA) operating with high vacuum, at an accelerating voltage of 20 kV and coupled to an energy-dispersive X-ray spectroscope (EDS, JEOL, JSM-6010LA).

#### X-Ray Diffraction (XRD)

X-ray diffraction (XRD) measurements were performed using a STADI-P (Stoe^®^, Darmstadt, Germany) diffractometer coupled to a Mythen 1K (Dectris^®^, Baden, Switzerland) detector that collected X-ray photons. Data were recorded at room temperature with powdered samples, in the 2θ range from 5.0° to 64.265°, 50 kV, 40 mA and using MoKα1 (λ = 0.7093 Å). Peaks were identified using published and standardized structures from the Inorganic Crystal Structure Database (ICSD).

#### X-Ray Photoelectron Spectroscopy (XPS)

X-ray photoelectron spectroscopy (XPS) measurements were performed using an X-ray photoelectron spectrometer (Thermo K-alpha spectromether, MA, United States) with a 72 W monochromated Al K-alpha^+^ source (*E* = 1486.6 eV) using 3000 eV, medium current, a spot size of 400 μm and 10 nm depth. Analyses were performed in two different points of powdered samples and the elemental composition was analyzed using CasaXPS software.

### Assays of Microbial H_2_S, MeSH, DMS, and DMDS Production

To measure H_2_S, MeSH, DMS, and DMDS production, *Pseudomonas* strains were grown overnight at 28°C in M9 minimal medium. Cultures were then adjusted to an OD_600_ = 0.3 and diluted 10-fold into 2 mL vials containing 300 μL of M9 medium supplemented with 0.25, 0.5, 1, 2, and 4 mM sulfate, Cys and Met as sole sulfur sources. Samples were incubated at 28°C for 48 h before measuring VSCs by gas chromatography using a flame photometric detector (Agilent 7890A GC fitted with a 7693 autosampler) and a HPINNOWax 30 m × 0.320 mm capillary column (Agilent Technologies J&W Scientific). An eight-point calibration curve of H_2_S, MeSH, DMS and DMDS standards were used as described in Carrión et al. (2015). Protein content in the cells was determined by the Bradford method (BioRad). Production of H_2_S, MeSH, DMS and DMS was expressed as mmol per mg protein.

To estimate the production of VSCs under nanoparticle biosynthesis conditions, cells were adjusted to an OD_600_ = 0.8 and inoculated into 300 μL of M9 medium supplemented with 2 mM sulfate, Cys or Met in the presence or absence of CdCl_2_ (20 μg mL^–1^). Samples were then incubated for 1, 24 and 48 h at 28°C in 2 mL sealed vials before quantifying the VSCs produced by gas chromatography as above.

### Data Analysis

Standard deviations (SD) of the results were expressed as mean (±). Statistical analysis of VSC production was carried out using GraphPad Prism 6.0 (GraphPad Software, Inc.). Error bars represent SD (*n* = 3). Student’s *t*-test was performed considering *p* < 0.05. Statistical significance was indicated as follows: ^*^p < 0.05, ^∗∗^*p* < 0.01 and ^∗∗∗^*p* < 0.001; ns, not significant.

## Results

### QDs Biosynthesis and Hydrogen Sulfide Production

**FIGURE 1:**
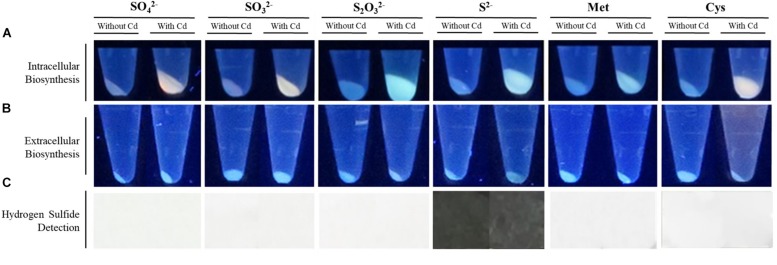
Biosynthesis of CdS QDs by *P. fragi* CG01 and hydrogen sulfide (H_2_S) production in the presence of different sulfur sources at 28°C. **(A)** Fluorescence of bacterial pellets exposed to biosynthesis conditions (CdCl_2_ 10 μg mL^–1^) using 0.25 mM SO_4_^2–^, SO_3_^2–^, S_2_O_3_^2–^, S^2–^, Met or Cys as sole sulfur sources after 48 h incubation. **(B)** Fluorescence of bacterial supernatant after UV light exposure. *P. fragi* CG01 was exposed to biosynthesis conditions with SO_4_^2–^, SO_3_^2–^, S_2_O_3_^2–^, S^2–^, Met or Cys as sole sulfur sources at 0.5 mM for 2 h. **(C)** Production of H_2_S by *P. fragi* CG01 was observed as a dark precipitate on white papers treated with a lead acetate solution. Cells were grown with 0.5 mM of SO_4_^2–^, SO_3_^2–^, S_2_O_3_^2–^, S^2–^, Met or Cys as sole sulfur sources for 2 h in minimal medium, in the presence or absence of CdCl_2_ (10 μg mL^–1^).

*Pseudomonas fragi* GC01 was able to use SO_4_^2–^, SO_3_^2–^, S^2–^, S_2_O_3_^2–^, Met and Cys as sole sulfur sources, although maximal growth was obtained with the former (data not shown). This suggests that these molecules could act as substrates to generate the S^2–^ required for the synthesis of CdS nanoparticles. To further investigate this hypothesis, the ability of *P. fragi* GC01 to biosynthesize CdS QDs from different S-containing molecules was evaluated. After 48 h exposure to biosynthetic conditions (0.25 mM sulfur source and 10 μg mL^–1^ CdCl_2_), *P. fragi* GC01 cell pellets showed fluorescence when excited with UV light (λ = 360 nm) (Figure 1A). As previously reported, the generation of fluorescent pellets in cells exposed to Cd is evidence of intracellular production of CdS QDs (Monrás et al., 2012; Gallardo et al., 2014; Plaza et al., 2016). A slightly fluorescent supernatant was only observed in the presence of Cys, indicating that extracellular QDs biosynthesis occurred. Also, this result suggests that no VSCs involved in the extracellular production of CdS are generated by cells under the other conditions tested (Figure 1B).

In general, most biosynthesis methods described to date require sulfur-containing molecules with high affinity for Cd^2+^ such as antioxidant thiols or the VSC H_2_S (Holmes et al., 1997; Bai et al., 2009b; Monrás et al., 2012; Gallardo et al., 2014). However, as reported in our previous work, QDs biosynthesis by *P. fragi* GC01 on LB media were not directly related to the production of H_2_S (Gallardo et al., 2014). Based on this, we studied the production of H_2_S by *P. fragi* GC01 and obtained results revealed that no H_2_S is generated when SO_4_^2–^, SO_3_^2–^, S_2_O_3_^2–^, Met or Cys are used as sulfur sources (Figure 1C). As expected, H_2_S was only observed when *P. fragi* GC01 cultures were exposed to S^2–^, probably as a consequence of the volatilization of sulfide from the medium as a consequence of bacterial activity.

### Extracellular Biosynthesis of CdS QDs

**FIGURE 2:**
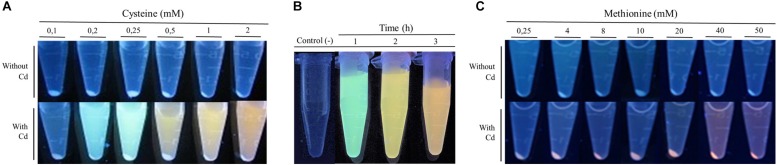
Fluorescence of *P. fragi* CG01 supernatants under biosynthesis conditions (CdCl_2_ 20 μg mL^–1^) after UV light exposure as indication of extracellular biosynthesis of Cds QDs. **(A)** Biosynthesis in minimal medium from various Cys concentrations in the presence or absence of CdCl_2_ (20 μg mL^–1^) after 2 h of incubation. **(B)** Fluorescence of bacterial supernatants of cells exposed to biosynthesis conditions (Cys 2 mM and CdCl_2_ 20 μg mL^–1^) at different incubation times. **(C)** Fluorescence of bacterial supernatant of *P. fragi* CG01 grown in minimal medium with various concentrations of Met in the presence or absence of CdCl_2_ (20 μg mL^–1^) for 72 h.

Based on the result obtained regarding the effect of Cys in the extracellular biosynthesis of CdS QDs by *P. fragi* GC01 (Figure 1B), we decided to evaluate new biosynthesis conditions. Extracellular biosynthesis of CdS QDs was observed in the presence of CdCl_2_ (20 μg mL^–1^) and Cys at concentrations ranging from 0.1 to 2 mM (Figure 2A). The intensity of the fluorescence in the supernatants indicated that the best condition for extracellular CdS QDs biosynthesis was 2 mM Cys and 20 μg mL^–1^ CdCl_2_ (Figure 2A). Using this condition, we evaluated the biosynthesis of QDs by *P. fragi* GC01 at different times to study the generation of different emission colors, a unique characteristic of QDs associated with time-dependent nanocrystal growth (Ulloa et al., 2016; Venegas et al., 2017; Bruna et al., 2019). Green, yellow and orange fluorescence colors were observed in culture supernatants of *P. fragi* GC01, confirming the extracellular generation of CdS QDs under this condition (Figure 2B).

In addition, we evaluated the effect of SO_4_^2–^, SO_3_^2–^, S_2_O_3_^2–^ or Met on the extracellular QDs biosynthesis by *P. fragi* GC01 in the presence of CdCl_2_ (20 μg mL^–1^) and using different concentrations of the S-sources (0.25–50 mM). Fluorescence was only observed in the supernatants of cultures incubated with 40 and 50 mM Met. Supernatants of these cultures were red after 72 h incubation (Figure 2C). However, fluorescent bacterial pellets were observed at all Met concentrations tested, suggesting the formation of intracellular QDs under these conditions (Figure 2C).

### Characterization of Biosynthesized CdS QDs

Quantum dots produced in supernatants of *P. fragi* GC01 cultures exposed to Cys and Met were purified and characterized. Specifically, the stability (based on the zeta potential) and the average hydrodynamic size of biosynthesized QDs were assessed by DLS. Biosynthesized QDs obtained with Cys had PDI values ranging from 0.50 to 0.53 and an average zeta potential (mean ± standard deviation) from −20.67 ± 0.64 mV to −15.70 ± 3.18, whereas nanoparticles synthesized using Met as sole sulfur source presented a PDI of 0.38 ± 0.04 and a zeta potential of −33.57 ± 2.76 mV. Moreover, the average nanocrystal size was below 36 nm in biosynthesis conditions with Cys (2 mM) and Met (40 mM) (Table 1).

**TABLE 1:** Particle size, PDI and Zeta potential of CdS QDs biosynthesized by *P. fragi* GC01 at different incubation times with cysteine (Cys) and after 72 h incubation using methionine (Met) as sole sulfur source.

**Nanoparticles**	**Particle size (nm)**	**Polydispersity index (PDI)**	**Zeta potential (mV)**
Cys - 1 h (green)	35.48 ± 4.96	0.53 ± 0.02	−15.70 ± 3.18
Cys - 2 h (yellow)	24.40 ± 5.09	0.52 ± 0.01	−20.23 ± 3.94
Cys - 3 h (orange)	27.39 ± 2.72	0.50 ± 0.05	−20.67 ± 0.64
Met - 72 h (red)	27.71 ± 3.02	0.38 ± 0.04	−33.57 ± 2.76

*Data are presented as mean ± standard deviation.*

**FIGURE 3:**
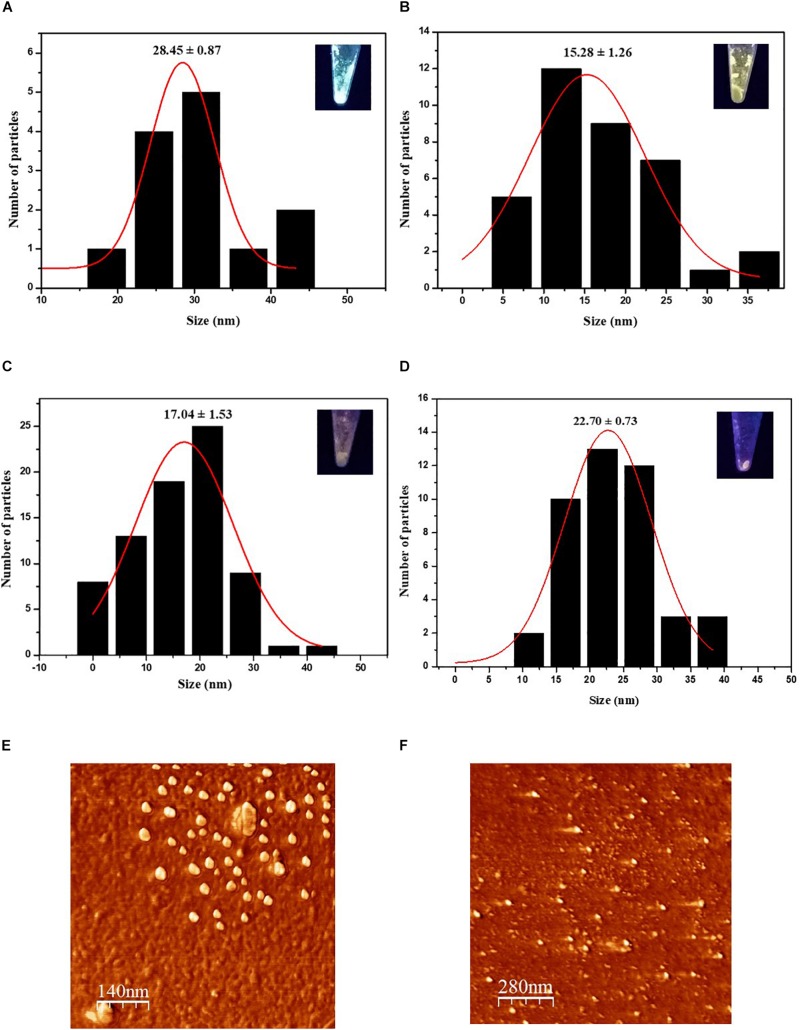
Characterization of CdS nanoparticles biosynthesized by *P. fragi* GC01 by AFM. AFM histogram of average solid-state size of nanoparticles synthesized from Cys showed **(A)** green, **(B)** yellow and **(C)** orange fluorescence **(D)**. AFM histogram of average solid-state size of nanoparticles synthesized with Met. 2D AFM topographical image of CdS nanoparticles synthesized with Cys for 3 h (orange) **(E)** and Met after 72 h **(F)**.

**FIGURE 4:**
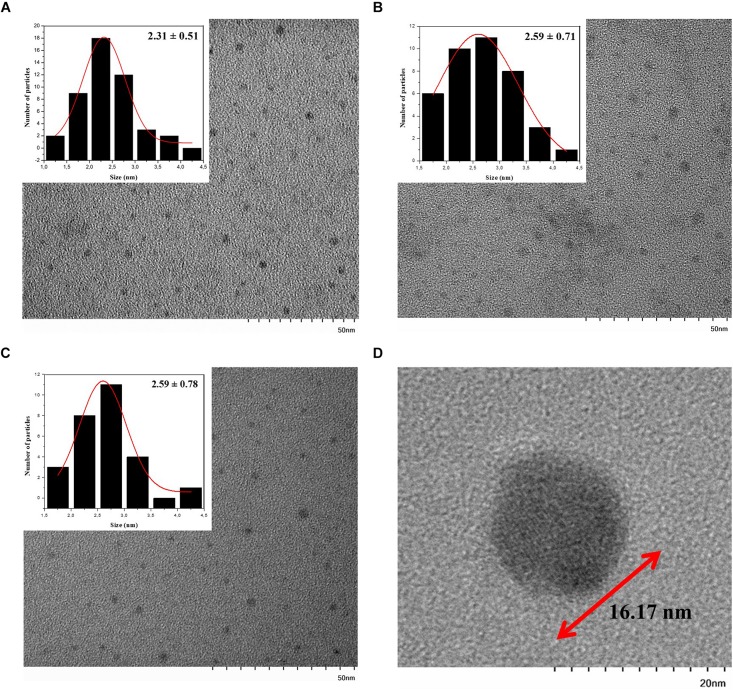
TEM image of CdS QDs biosynthesized with Cys or Met (inset: size histogram). QDs produced in presence of Cys and exhibiting **(A)** green **(B)** yellow and **(C)** orange fluorescence were evaluated. **(D)** CdS QDs biosynthesized with Met.

In addition to the hydrodynamic size, a quantitative size analysis of the biosynthesized CdS QDs was implemented with atomic force microscopy (AFM) and transmission electron microscopy (TEM). The AFM topological images of nanoparticles synthesized with Cys and Met as sole sulfur source were analyzed (Figures 3E,F). This technique allowed us to evaluate the average size of biosynthesized nanoparticles in solid-state. The average diameters of QDs produced in the presence of Cys were 28.45 ± 0.87, 15.28 ± 1.26, and 17.04 ± 1.53 nm for green, yellow and orange nanoparticles, respectively, (Figures 3A–C), while the average diameter of QDs obtained with Met was 22.70 ± 0.73 nm (Figure 3D). The QDs with green fluorescence color, corresponding to the nanoparticles obtained after 1 h of synthesis (Figure 3A), were larger than the rest of the QDs, despite being obtained in a shorter time of synthesis and their fluorescence color corresponding to smaller nanoparticle sizes. This could be due to the high polydispersity index (PDI) of the biosynthesized nanoparticles (Table 1) probably as consequence of the organic cover, in addition to the low stability of the green nanoparticles, causing their agglomeration. TEM analysis of CdS nanoparticles revealed that the nanoparticles produced by *P. fragi* GC01 had a spherical-like morphology with a homogenous size distribution (Figure 4). TEM images determined that the average sizes of QDs produced in the presence of Cys were 2.31 ± 0.51, 2.59 ± 0.71, and 2.59 ± 0.78 nm for green, yellow and orange fluorescent nanoparticles, respectively, (Figures 4A–C). The diameter of QDs prepared with Met was ∼16 nm, and showed the presence of planes, evincing the presence of a crystalline structure (Figure 4D). The small size of the nanoparticles obtained with Cys (∼2 nm) did not show clear differences between green, yellow and orange nanoparticles. However, TEM analysis confirmed the formation of QD-type nanoparticles with a size below 20 nm (Rengers et al., 2019).

**FIGURE 5:**
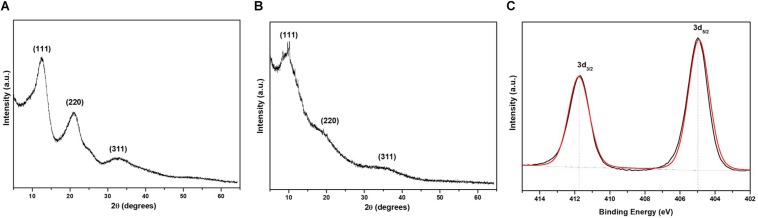
X-ray diffraction and X-ray photoelectron spectroscopy of QDs biosynthesized by *P. fragi* GC01. XRD pattern of CdS nanoparticles synthesized in cultures in the presence of **(A)** Cys and **(B)** Met as sole sulfur sources. **(C)** XPS spectra of Cd 3d region of CdS QDs obtained with Cys.

X-ray diffraction measurements were performed to evaluate the crystal nature of CdS QDs. Figures 5A,B show the XRD patterns for CdS nanoparticles prepared with Cys and Met. Three characteristic peaks were observed at 12.85°, 21.06°, and 32.69°, respectively, (111, 220 and 311 planes, indexed as cubic CdS). Bai et al. (2009a, b) obtained similar results for CdS nanoparticles synthesized by *Rhodobacter sphaeroides* and *Rhodopseudomonas palustris* (Bai et al., 2009a, 2009b). Additionally, defined peaks were observed for CdS nanoparticles obtained with Cys (Figure 5A). Conversely, nanoparticles produced in the presence of Met displayed broader peaks, indicating defects in the crystal and a larger quantity of amorphous material, suggesting a thicker organic coating (Figure 5B). This could be consequence of high amounts of organic molecules attached to the nanoparticle surface, resulting in a crystal-amorphous interfacial effect (Muntaz Begum et al., 2016).

X-ray photoelectron spectroscopy is a measurement of the surface of the sample, able to access only a depth of 10 nm. Spectra of CdS QDs produced from Cys and Met were evaluated with this technique. Survey spectra of CdS nanoparticles biosynthesized with Cys presented C 1s, Cd 3d, Na 1s, O 1s, P 2p, and S 2p (Supplementary Figure S1A). Cd 3d deconvoluted peaks (Figure 5C) indicated binding energies of 412 and 405 eV, corresponding to Cd_3__/__2_ and Cd_5__/__2_ as reported by Marusak et al. (2016) and Richards et al. (2016). Survey spectra of CdS obtained from Met contained similar elements, but it was not possible to identify Cd 3d peaks (Supplementary Figure S1B). This is probably due to the thick organic surface coating, which avoids Cd detection by XPS. Despite XPS not allowing the identification of Cd in QDs biosynthesized in the presence of Met, XRD confirmed the formation of cubic CdS QDs and indicated the presence of amorphous coating on nanoparticles synthesized from Met (Figure 5B).

**FIGURE 6:**
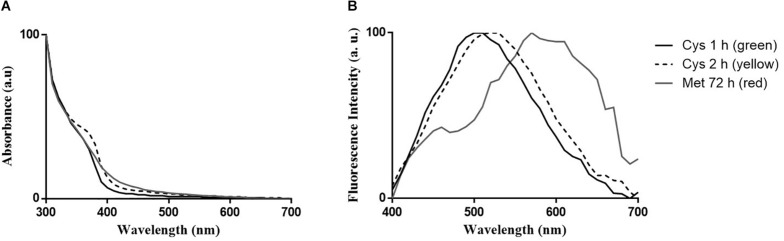
Optical properties of CdS QDs biosynthesized by *P. fragi* GC01. **(A)** UV-vis absorption spectra of purified QDs and **(B)** fluorescence emission spectra (λ_excitation_ = 360 nm).

Spectroscopic properties of nanoparticles produced by *P. fragi* GC01 after 1 and 2 h of incubation under biosynthesis conditions with Cys (green and yellow nanoparticles, respectively) and after 72 h of incubation with Met (red nanoparticles) were evaluated. The absorbance spectra of the purified nanoparticle fractions showed a peak with maximum absorption at 360 nm for green nanoparticles, 370 nm for yellow nanoparticles and 380 nm for red nanoparticles (Figure 6A). This is in agreement with previous reports of biosynthesized CdS nanoparticles (Mi et al., 2011; Yang et al., 2015; Plaza et al., 2016; Bruna et al., 2019). Regarding the emission spectra of purified samples, emission peaks between 470 and 530 nm, 490 and 550 nm and 550 and 620 nm were determined for green, yellow and red nanoparticles, respectively, (Figure 6B). As expected, different emission spectra were observed for Cys green (1 h) and yellow (2 h) CdS QDs, with maximum fluorescence peaks at 500 and 530 nm, respectively, (Figure 6B). Additionally, red nanoparticles (Met 72 h) showed an emission spectrum with maximum fluorescence peaks at 570 nm (Figure 6B). The quantum yields (QY) of the CdS QDs biosynthesized from Cys and Met were obtained by comparison with the QY of fluorescein in ethanol (standard). The quantum yields for the CdS QDs with Cys (2 h) and Met (72 h) were 21.04 and 7.81%, respectively.

The composition of biosynthesized nanoparticles was determined by EDS. QDs obtained after 3 h (orange) of biosynthesis with Cys (Supplementary Figure S2) showed Cd and sulfur (S) element signals. In addition, signals corresponding to oxygen (O) and carbon (C) were also detected, probably as part of the organic cover of QDs. It should be noted that C signal could come from both the coating of the nanoparticle or from the carbon tape used in the SEM sample holder. This result was observed in all the nanoparticles biosynthesized in the presence of Cys (Supplementary Figure S2).

### Production of VSCs by *P. fragi* GC01

**FIGURE 7:**
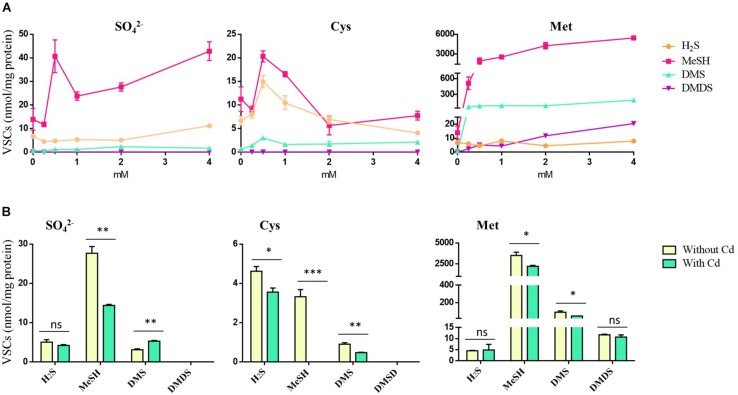
VSCs produced by *P*. *fragi* GC01 from SO_4_^2–^, Cys and Met under biosynthesis conditions. **(A)** VSC production from sulfur sources with concentrations ranging from 0 to 4 mM after 48 h of incubation. **(B)** VSC production under biosynthesis conditions with different sulfur sources in the presence or absence of CdCl_2_ (20 μg mL^–1^) after 48 h. Results represent the average of three biological replicates with their respective standard deviations. Student’s *t*-test (*P* < 0.05): Comparison between treatments with and without cadmium (Cd). Statistically significant differences are shown as: ^∗∗∗^*P* < 0.001, ^∗∗^*P* < 0.01, ^*^P < 0.05.

*Pseudomonas fragi* GC01 was able to produce intracellular CdS QDs when grown on different sulfur compounds and extracellular QDs when Cys and Met were used as sole sulfur sources (see above). However, it was not possible to relate the formation of CdS nanoparticles to the production of H_2_S under the conditions tested. Therefore, to elucidate the source of S^–2^ involved in QD biosynthesis by *P. fragi* GC01, we analyzed the VSCs produced by this strain. Specifically, the ability of *P. fragi* GC01 to release H_2_S, MeSH, DMS and DMDS was evaluated when SO_4_^2–^, Cys or Met were used as sole sulfur source for bacterial growth and CdS biosynthesis. As shown in Figure 7A, *P. fragi* GC01 produced H_2_S, MeSH and DMS in the presence of SO_4_^2–^, Cys and Met, with concentrations of MeSH between 2- to 600-fold and 6- to 28-fold higher than those of H_2_S and DMS, respectively. Moreover, cells grown with sulfate or Cys produced low levels of VSCs, even at high concentrations of substrate (below 42 mmol per mg protein) (Figure 7A). Finally, maximal formation of DMS and MeSH was observed when Met was used as sole sulfur source (190 and 5400 mmol per mg protein), and this was the only condition in which DMDS was detected (Figure 7A).

The production of VSCs by *P. fragi* GC01 under QDs biosynthesis conditions (presence of sulfur source 2 mM and 20 μg mL^–1^ CdCl_2_) after 48 h of incubation was also tested (Figure 7B). This selection was based on the ability to biosynthesize extracellular QDs when this strain was supplemented with Cys (2 mM) after 1 h, and intracellular biosynthesis from Met and SO_4_^2–^ (2 mM) after 48 h. Low levels of H_2_S were determined in all sulfur sources analyzed, and a decrease in H_2_S was observed after Cd exposure (biosynthesis conditions) when Cys was used as a sulfur source (Figure 7B). MeSH production from all sulfur compounds decreased in the presence of Cd, suggesting that this gas could be a source of S^2–^ that interacts with Cd^2+^. In addition, DMS levels decreased in incubations with Cd and the sulfur sources Cys or Met. Finally, no statistically significant differences (ns, *P* > 0.05) in DMDS production were determined between treatments with and without Cd (Figure 7B). These results suggest that H_2_S and MeSH are involved in the generation of CdS QDs, providing directly or indirectly the S^2–^ that interacts with Cd^2+^ to form the nanocrystal seed.

### VSC Catabolic Pathways Associated to Biosynthesis of CdS QDs in *Pseudomonas deceptionensis*

*Pseudomonas deceptionensis* M1^T^ was used to study the possible relationship between nanoparticle biosynthesis and VSC catabolism, since its genome has been sequenced and the pathways of DMS and MeSH production elucidated (Carrión et al., 2015). Specifically, Carrión et al. (2015) showed that MeSH was synthesized in this strain from Met in a reaction catalyzed by methionine gamma lyase (encoded by *megL*). In turn, MeSH was converted into DMS *via* a methyltransferase termed MddA. *P. deceptionensis* M1^T^ wild type, *P. deceptionensis* M1^T^ *megL*^–^ (unable to produce MeSH from Met) and *P. deceptionensis* M1^T^ *mddA*^–^ (unable to synthesize DMS from MeSH) (Carrión et al., 2015) were used to study the production of extra- and intracellular nanoparticles in the presence of Cys or Met, using the same conditions as in *P. fragi* GC01 (Cys or Met 2 mM and CdCl_2_ 20 μg mL^–1^). Extracellular biosynthesis was detected in the three *P. deceptionensis* M1^T^ strains after 1 h incubation with Cys (Supplementary Figure S3A). As expected, all the strains produced H_2_S, although lower concentrations were observed in the presence of Cd^2+^ (Supplementary Figure S3B). These results suggest that QDs production is mediated by H_2_S as it has been reported in other bacterial strains (Bai et al., 2009a; Ulloa et al., 2016; Bruna et al., 2019). Finally, *P. deceptionensis* M1^T^ produced higher levels of H_2_S than *P. fragi* GC01 (Supplementary Figure S3), a phenomenon that could be explained by the presence of Cys desulfhydrase, which converts Cys into H_2_S.

**FIGURE 8:**
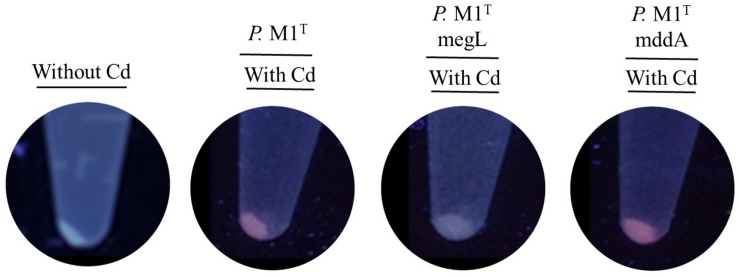
Biosynthesis of CdS QDs by *P. deceptionensis* M1^T^ strains in the presence of Met as sole sulfur source. Fluorescence of supernatants of *P. deceptionensis* M1^T^ strains under biosynthesis conditions after UV light exposure. Bacterial strains were grown in M9 medium with 2 mM Met, in presence or absence of CdCl_2_ 20 μg mL^–1^ for 48 h.

The results shown in Figure 8 suggest that QDs biosynthesis from Met is mainly associated to the production of MeSH in *P. deceptionensis* M1^T^ wild type and *mddA*^–^ strains. In support of this hypothesis, *P. deceptionensis* M1^T^ *megL*^–^, which does not produce MeSH from Met (Carrión et al., 2015), did not generate fluorescent nanoparticles or pellets under biosynthesis conditions (Figure 8). Finally, *P. deceptionensis* M1^T^ *mddA*^–^, although it does not synthesize DMS, was able to produce CdS QDs in the presence of Met, discarding DMS as a substrate for nanoparticle biosynthesis (Figure 8).

## Discussion

The use of bacteria as cell factories to produce nanoparticles with great economic and technological value has increased in the past few years as a safe and eco-friendly alternative, but it also provides the possibility to manufacture nanoparticles with new properties and applications. In a previous work, we reported the use of different Antarctic *Pseudomonas* spp., resistant to oxidative stress, to biosynthesize CdS QDs (Gallardo et al., 2014). The synthesis of CdS QDs was intracellular and it was performed at low temperatures (15°C). However, it was not possible to associate the CdS nanoparticles biosynthesis in *P. fragi* GC01 to the use of sulfur-containing molecules with affinity to Cd as a precursor to their biosynthesis, such as antioxidant thiols and volatile sulfur compounds as H_2_S (Holmes et al., 1997; Bai et al., 2009b; Monrás et al., 2012; Gallardo et al., 2014). Consequently, we decided to study the ability of *P. fragi* GC01 to use different sulfur sources to grow, to biosynthesize CdS QDs and to produce VSCs.

In this work, we study the link between CdS QDs biosynthesis and production of VSCs from different sulfur sources, focusing on Cys and Met, in *P. fragi* GC01. We showed that this strain can grow on a wide variety of sulfur sources such as sulfate, sulfite, sulfide, thiosulfate, Met and Cys, as well as produce intracellular nanoparticles from them. Sulfur is an essential element for cell growth, but it can only be assimilated as S^2–^ in its fully reduced state. The sulfur required by bacteria can be obtained in both inorganic and organic forms (Kertesz, 2001). Inorganic sulfate is the most abundant sulfur source in the environment and the main metabolic pathway by which bacteria assimilate this element (Kertesz, 2001). Sulfate assimilation occurs by active transportation of the substrate into the cell by an ABC-type transporter and is subsequently reduced to S^2–^ before being assimilated into organic material (Kertesz, 2000, 2004). However, bacteria can also use other inorganic sulfur sources to grow such as sulfite, thiosulfate and sulfide. Sulfite and sulfide can enter the sulfate assimilation pathway, where sulfite is reduced to sulfide by the enzyme sulfite reductase (Kredich, 2008). Moreover, thiosulfate is incorporated into *Pseudomonas* cells through an ABC-type transporter for sulfate/thiosulfate (Kertesz, 2004) and can be assimilated after being reduced to sulfide by the enzyme thiosulfate reductase (Barton et al., 2017). In addition, Cys can be assimilated directly by bacteria *via* a transsulfuration pathway with subsequent formation of Met, or can be used by cysteine desulfhydrase to produce sulfide (Awano et al., 2003, 2005). Finally, Met can be used as sole sulfur source by some bacteria. Met may be provided to the cells by desulfurization to yield inorganic sulfate or by a reverse transsulfuration pathway, which converts Met to Cys. This pathway has been reported in bacteria from the *Pseudomonas* genus (Vermeij and Kertesz, 1999).

In the study of the intracellular biosynthesis of CdS nanoparticles from different sulfur sources by *P. fragi* GC01, Cd^+2^ was exogenously provided as a substrate, with bacterial metabolism being responsible for providing the S^–2^ needed for the formation of CdS QDs. However, several metabolic pathways can be involved in the synthesis of CdS QDs through the generation of S^–2^, due to its incorporation into organic material as the final product of sulfur assimilation metabolism in bacteria (Kertesz, 2001, 2004; Awano et al., 2005; Kredich, 2008; Barton et al., 2017). No H_2_S was produced by *P. fragi* GC01 at detectable levels under any condition tested. Although H_2_S could be formed at concentrations below the detection limit, it is also possible that the absence of this VSC in *P. fragi* GC01 samples could be due to the lack or reduced activity of enzymes involved in the reduction of sulfur compounds to sulfide.

Conversely, extracellular biosynthesis of CdS QDs was only observed when Cys and Met were used as sole sulfur sources. The nanoparticles obtained with Cys showed several fluorescent colors of the supernatants (between green and orange) depending on the incubation time and Cys concentrations. This optical property is a unique and characteristic phenomenon of QDs, which emit different colors of fluorescence according to the nanocrystal size (Yang et al., 2016; Ulloa et al., 2018; Bruna et al., 2019). Nevertheless, the optical properties of fluorescence emission also can be affected by the surface defects decorating the nanoparticles, as has been reported by Bruna et al. (2019) in the biosynthesis of QDs of CdS by halophilic bacteria. Additionally, bacterial supernatants incubated with Met showed red fluorescence, the color of which did not vary over time. This observation strongly suggests that the nanoparticles produced by the extracellular biosynthesis of CdS QDs, when using Met as the sole sulfur source, could have different properties from the nanoparticles produced from Cys either because the S^2–^ as precursor of the QDs biosynthesis could be provided by different bacterial metabolic pathways or because other reduced sulfur anions are involved in CdS QDs biosynthesis when interacting with Cd.

Therefore, in this study we have established that *P. fragi* GC01 biosynthesize CdS QDs from Cys and Met through several characterization methods. CdS nanoparticles produced by this Antarctic strain showed a zeta potential value (lower than −20 mV) indicative of high stability in aqueous solutions, with nanocrystals produced with Met being the most stable (Kuznetsova and Rempel, 2015). The average size of the nanoparticles produced with Cys (∼2 nm) and Met (∼16 nm), determined from TEM images, were consistent with the size of QDs biosynthesized by other microorganisms (Chen et al., 2009; Khachatryan et al., 2009; Syed and Ahmad, 2013; Wu et al., 2015; Al-Shalabi and Doran, 2016; Tandon and Vats, 2016; Wang et al., 2018; Bruna et al., 2019). TEM as a technique for characterizing nanomaterials enables the visualization of the shape and size of the nanoparticles by providing direct images of nanomaterials at a spatial resolution (Lina et al., 2014). The results obtained with TEM showed mainly the diameter of the core (Kale et al., 2013), which explains the great discrepancy in the particle sizes observed between TEM (size < 17 nm), AFM (size in the range of 15–29 nm) and DLS (size in the range of 24–36 nm), because the latter two techniques might be considering the organic layer of the nanoparticles as well as presenting a greater aggregation of particles in the samples. In this context, the average hydrodynamic size of the nanoparticles estimated by DLS was larger than in AFM. DLS is based on the particle behavior in aqueous medium and hydrodynamic size is measured through Brownian motion, which considers both the size of the particle and the solvation layer, resulting in larger average diameters. On the other hand, AFM is commonly used to determine particle size distribution in solid state by the interaction between the sample and tip, where the tip is either repelled or attracted. Thus, AFM average diameter tends to present lower values when compared to DLS, as it represents only the particle size and does not consider the solvating layer (Hoo et al., 2008). The elemental analysis, the crystalline shape and surface composition of the nanoparticles by EDS, XRD and XPS confirm the formation of CdS QDs. Specifically, EDS analysis of the nanoparticles obtained from Cys showed signals associated to Cd and S elements, while the XRD taken from the nanoparticles synthesized from Cys and Met as sole sulfur source, indicated the formation of cubic nanocrystals of CdS, which are related to the diffraction of the crystalline planes (111), (220), and (311). The same XRD pattern has been reported for the CdS QDs obtained *via* chemical (Waly et al., 2017; Bharti et al., 2018; Wang et al., 2018) and biological synthesis (Sankhla et al., 2016; Chakraborty et al., 2018). XPS analysis was developed in the 1960 by Siegbahn’s research group and, since then, it has been widely used to determine the chemical composition and state of elements present on a sample surface (Siegbahn et al., 1967). To perform the measurements, an external X-ray beam is injected, commonly reaching 10 nm depth on the sample. The incident X-ray is able to eject electrons from core levels (1s, 2s, 2p…), and these photoelectrons have characteristic Kinect energies, which enables the differentiation of each element and their respective chemical state (Baer and Engelhard, 2010). In this work, the XPS analysis was carried out to study the surface composition of the nanocrystals. The XPS analysis of the surface composition of the nanocrystals produced with Cys and Met, presented signals in the C 1s, Cd 3d, Na 1s, O 1s, P 2p and S 2p region. The presence of N and S suggest that Cys and Met are part of the organic coating of the QDs surface, indicating that these sulfur compounds could be stabilizing the QDs. The Cd 3d spectrum deconvoluted peaks indicated binding energies of 412 and 405 eV, corresponding to Cd_3__/__2_ and Cd_5__/__2_, respectively, (Marusak et al., 2016; Richards et al., 2016). Furthermore, the intensity ratio of the Cd_3__/__2_ and Cd_5__/__2_ peaks was 2:3, confirming that Cd was obtained in the state of Cd^2+^, which is in accordance to what has been previously reported for CdS QDs (Bag et al., 2017). It was not possible to identify the Cd 3d peaks in the spectra of CdS QDs produced using Met, despite similar elements having been observed on the surface composition of the nanocrystals. This could be due to the different organic compounds of a thick (>10 nm) surface coating of the nanoparticle produced with Met, whereby the Cd may not be detected in this technique. Additionally, the presence of an amorphous coating on the nanoparticles obtained with Met was also determined by XRD (Muntaz Begum et al., 2016), a technique that confirmed the formation of cubic CdS QDs.

The absorbance spectra of green and yellow CdS nanoparticles produced with Cys and red nanoparticles from Met synthesized by *P. fragi* GC01 showed peaks at 360, 370 nm and 380, respectively. This is in agreement with the typical characteristic of CdS QDs, a plasmon resonance absorption with maximum absorption below 400 nm (Liu et al., 2014), in comparison with the absorption of bulk CdS nanoparticles at 515 nm (Song et al., 2014). The corresponding fluorescence emission peaks were observed at 500, 530 and 570 nm for green, yellow and red nanoparticles, respectively. Both peaks (absorbance and fluorescence) of QDs obtained from Cys progressively shifted to longer wavelengths over time, changing from green (1 h) to yellow (2 h) fluorescence color. This phenomenon can be explained by the different sizes of the purified nanoparticles (Yang et al., 2015; Dunleavy et al., 2016). The absorbance and fluorescence properties are in agreement with previous reports of biological synthesis of CdS nanoparticles, where the nanoparticles display maximal absorbance peaks between 360 and 380 nm and fluorescence emission peaks between 470 and 600 nm (Gallardo et al., 2014; Yang et al., 2015; Dunleavy et al., 2016; Plaza et al., 2016; Ulloa et al., 2016; Venegas et al., 2017; Glatstein et al., 2018). In addition, the quantum yields for both nanoparticles solutions obtained from Cys (yellow) and Met (red) were 21.04 and 7.81%, respectively. Interestingly, a high quantum yield (21.04%) was determined in QDs produced from Cys (Venegas et al., 2017; Bruna et al., 2019) in comparison to those generated from biosynthesis with Met. These results could be due to the size differences observed between nanoparticles, where the nanoparticles obtained from Cys (yellow) have a size of ∼2 nm and the nanoparticles from Met (red) have a size of ∼16 nm. Nevertheless, quantum yield (7.81%) determined in QDs produced from Met are high when compared to other reported biological syntheses (Yang et al., 2015; Jang et al., 2015; Al-Shalabi and Doran, 2016).

In this study, we have also shown that *P. fragi* GC01 can produce H_2_S, MeSH and DMS in the presence of SO_4_^2–^, Cys and Met. However, DMDS was only detected when Met was used as sole sulfur source. It has been reported that many bacterial strains from different environments can release VSCs such as H_2_S, MeSH, DMS, and DMDS among others, as a result of a sulfur assimilation process involved in the synthesis of amino acids or from biological degradation of sulfur-containing compounds (Lomans et al., 2002; Schulz and Dickschat, 2007; Korpi et al., 2009). Specifically, Met yielded the highest concentrations of MeSH and DMS, although it generated low concentrations of DMDS and H_2_S. MeSH is the main VSC released by *P. fragi* GC01 from all sulfur sources tested. Met has been described as a major precursor of VSC biosynthesis, especially of MeSH (Vermeij and Kertesz, 1999; Lu et al., 2013; Carrión et al., 2015), which, in turn, is a precursor of DMS and DMDS (Lu et al., 2013). The formation of MeSH from Met is catalyzed by methionine ɣ-lyase (Vermeij and Kertesz, 1999). Additionally, MeSH can be produced by methylation of H_2_S in a reaction catalyzed by a thiol S-methyltransferase enzyme. On the other hand, the production of DMS by methylation of MeSH is mediated by the methyltransferase MddA, which uses S-adenosyl-L-methionine (Ado-Met) as methyl donor (Carrión et al., 2015). However, DMDS can be produced by chemical oxidation of MeSH (Chin and Lindsay, 1994; Lu et al., 2013). Besides, Cys has been described as the main precursor of H_2_S, through a reaction catalyzed mainly by Cys desulfhydrase (Awano et al., 2003, 2005). Additionally, Cys can be incorporated into the cell directly *via* trans-sulfuration to form Met, which in turn is the precursor of the rest of the volatiles detected (Vermeij and Kertesz, 1999). When SO_4_^2–^ is used as sole sulfur source, it is reduced to sulfide (S^–2^), which leads to the production of Cys and Met (Kertesz, 2004).

Volatile sulfur compounds production by *P. fragi* GC01 under biosynthesis conditions (presence of Cd^+2^) with Cys and Met was tested. The main results obtained showed a significant statistical decrease of H_2_S between the treatment with and without Cd in presence of Cys (*P* < 0.05). By the other hand, the production of MeSH significatively decreased when Cd^+2^ (CdCl_2_) was added in presence of all sulfur sources tested (*P* < 0.05). Although the production of several VSCs has been reported in bacteria, only H_2_S has been associated with the biosynthesis of CdS nanoparticles (Bai et al., 2009a; Gallardo et al., 2014; Yang et al., 2016). The production of VSCs by *P. fragi* GC01 under biosynthesis conditions showed higher H_2_S and MeSH production from Cys and Met. In addition, a statistically significant reduction of both VSCs (*P* < 0.05) was observed when samples were treated with Cd. Decreased H_2_S production in the Cd treatment suggests that the production of QDs is mediated by H_2_S, as has been previously reported in other bacteria (Bai et al., 2009a; Yang et al., 2015). In general, different authors have suggested that the mechanism of cadmium-based nanoparticle formation was the generation of H_2_S (as sulfide source) from Cys in a reaction mediated by Cys desulfhydrase (Holmes et al., 1997; Bai et al., 2009a) or cystathionine γ-lyase (Dunleavy et al., 2016). Both enzymes catalyze the formation of pyruvate, ammonia and hydrogen sulfide from Cys. Therefore, the fluorescence in the bacterial supernatant indicated extracellular CdS QDs formation by the reaction of Cd^2+^ added into the system with S^2–^ provided by the bacterial metabolism, likely through the H_2_S production as a result of enzymatic degradation of Cys (Bai et al., 2009a; Yang et al., 2016). In addition to being a sulfur source, the amino acid Cys could also act as a capping agent in CdS nanoparticles formation (Dunleavy et al., 2016).

The importance of Met in the biosynthesis of CdS QDs and the role of VSCs’ catabolic pathways were studied using different strains of *P. deceptionensis* M1^T^. Carrión et al. (2015) described a novel pathway of DMS production from MeSH, which in turn is a product of Met catabolism. Intracellular biosynthesis of CdS QDs was observed in all *P. deceptionensis* M1^T^ strains except for *P. deceptionensis* M1^T^ *megL*^–^, which cannot form MeSH from Met. In addition, the fact that *deceptionensis* M1^T^ *mddA*^–^ was able to synthesize nanoparticles discards DMS as a substrate in this process, since this strain cannot produce DMS from MeSH. These results strongly suggest that MeSH could act as a source of S^2–^ to synthesize CdS QDs when Met is added to the medium as sole sulfur source.

## Conclusion

In conclusion, here we establish that *P. fragi* GC01 is able to biosynthesize extracellular CdS QDs in the presence of the amino acids Cys and Met. The bioproduction of these nanoparticles is linked to the ability of this strain to form the volatile sulfur compounds H_2_S and MeSH from Cys and Met, respectively. Both VSCs produced by *P. fragi* GC01 act as a source of S^2–^ in the biosynthesis of CdS QDs. Interestingly, the biosynthesis of Cd-based nanoparticles from Met has not been previously described, nor has the participation of volatile organic compounds such as MeSH in the biosynthesis process. Therefore, this study constitutes the first report correlating the production of the VSC MeSH and the biosynthesis of CdS QDs.

## Data Availability

The datasets generated for this study are available on request to the corresponding author.

## Author Contributions

CG-B, JP-D, and AQ conceived and designed the study, analyzed the data and prepared the manuscript. CG-B performed the experiments. JP contributed to performing the experiments of characterization of nanoparticles. JP, AS, ND, and OR analyzed the data and discussion regarding the characterization of nanoparticles. OC and JT designed, analyzed the data and discussion regarding the Volatile Sulfur Compounds. All authors commented on or contributed to the manuscript.

## Conflict of Interest Statement

The authors declare that the research was conducted in the absence of any commercial or financial relationships that could be construed as a potential conflict of interest.

## References

[B1] Ali J. Hameed A. Ahmed S. Ali M. I. (2016). Role of catalytic protein and stabilizing agents in transformation of Ag ions to nanoparticles by *Pseudomonas aeruginosa*. *IET Nanobiotechnol.* 10 295–300. 10.1049/iet-nbt.2015.0093 27676377 PMC8676123

[B2] Alivisatos A. P. (1996). Perspectives on the physical chemistry of semiconductor nanocrystals. *J. Phys. Chem.* 100 13226–13239. 10.1021/jp9535506

[B3] Al-Shalabi Z. Doran P. M. (2016). Biosynthesis of fluorescent CdS nanocrystals with semiconductor properties: comparison of microbial and plant production systems. *J. Biotechnol.* 223 13–23. 10.1016/j.jbiotec.2016.02.018 26880539

[B4] Awano N. Wada M. Kohdoh A. Oikawa T. Takagi H. Nakamori S. (2003). Effect of cysteine desulfhydrase gene disruption on L-cysteine overproduction in *Escherichia coli*. *Appl. Microbiol. Biotechnol.* 62 239–243. 10.1007/s00253-003-1262-2 12883870

[B5] Awano N. Wada M. Mori H. Nakamori S. Takari H. (2005). Identification and functional analysis of *Escherichia coli* cysteine desulfhydrases. *Society* 71 4149–4152. 10.1128/AEM.71.7.4149 16000837 PMC1169034

[B6] Ayano H. Kuroda M. Soda S. Ike M. (2015). Effects of culture conditions of *Pseudomonas aeruginosa* strain RB on the synthesis of CdSe nanoparticles. *J. Biosci. Bioeng.* 119 440–445. 10.1016/j.jbiosc.2014.09.021 25454693

[B7] Baer D. R. Engelhard M. H. (2010). XPS analysis of nanostructured materials and biological surfaces. *J. Electron Spectros. Relat. Phenomena* 17 415–432. 10.1016/j.elspec.2009.09.003

[B8] Bag P. P. Wang X. S. Sahoo P. Xiong J. Cao R. (2017). Efficient photocatalytic hydrogen evolution under visible light by ternary composite CdS@NU-1000/RGO. *Catal. Sci. Technol.* 7 5113–5119. 10.1039/c7cy01254c

[B9] Bai H. J. Zhang Z. Guo Y. Jia W. (2009a). Biological synthesis of size-controlled cadmium sulfide nanoparticles using immobilized Rhodobacter sphaeroides. *Nanoscale Res. Lett.* 4 717–723. 10.1007/s11671-009-9303-0 20596372 PMC2894101

[B10] Bai H. J. Zhang Z. M. Guo Y. Yang G. E. (2009b). Biosynthesis of cadmium sulfide nanoparticles by photosynthetic bacteria *Rhodopseudomonas palustris*. *Coll. Surfaces B Biointer.* 70 142–146. 10.1016/j.colsurfb.2008.12.025 19167198

[B11] Baker S. Mohan Kumar K. Santosh P. Rakshith D. Satish S. (2015). Extracellular synthesis of silver nanoparticles by novel *Pseudomonas veronii* AS41G inhabiting *Annona squamosa* L. and their bactericidal activity. *Spectrochim. Acta A Mol. Biomol. Spectrosc.* 136 1434–1440. 10.1016/j.saa.2014.10.033 25459703

[B12] Barton L. L. Ritz N. L. Fauque G. D. Lin H. C. (2017). Sulfur cycling and the intestinal microbiome. *Dig. Dis. Sci.* 62 2241–2257. 10.1016/j.asoc.2017.08.004 28766244

[B13] Bharti D. B. Bharati A. V. Wankhade A. V. (2018). Synthesis, characterization and optical property investigation of CdS nanoparticles. *Luminescence* 33 1445–1449. 10.1002/bio.3572 30378241

[B14] Bruna N. Collao B. Tello A. Caravantes P. Díaz-Silva N. Monrás J. P. (2019). Synthesis of salt-stable fluorescent nanoparticles (quantum dots) by polyextremophile halophilic bacteria. *Sci. Rep.* 9:1953. 10.1038/s41598-018-38330-8 30760793 PMC6374371

[B15] Carrión O. Curson A. R. J. Kumaresan D. Fu Y. Lang A. S. Mercadé E. (2015). A novel pathway producing dimethylsulphide in bacteria is widespread in soil environments. *Nat. Commun.* 6:6579. 10.1038/ncomms7579 25807229

[B16] Carrión O. Miñana-Galbis D. Montes M. J. Mercadé E. (2011). Pseudomonas deceptionensis sp. nov., a psychrotolerant bacterium from the antarctic. *Int. J. Syst. Evol. Microbiol.* 61 2401–2405. 10.1099/ijs.0.024919-0 21062736

[B17] Chakraborty J. Mallick S. Raj R. Das S. (2018). Functionalization of extracellular polymers of *Pseudomonas aeruginosa* N6P6 for synthesis of CdS nanoparticles and cadmium bioadsorption. *J. Polym. Environ.* 26 3097–3108. 10.1007/s10924-018-1195-6

[B18] Chen Y. L. Tuan H. Y. Tien C. W. Lo W. H. Liang H. C. Hu Y. C. (2009). Augmented biosynthesis of cadmium sulfide nanoparticles by genetically engineered *Escherichia coli*. *Biotechnol. Prog.* 25 1260–1266. 10.1002/btpr.199 19630084

[B19] Chin H. W. Lindsay R. C. (1994). Ascorbate and transition-metal mediation of methanethiol oxidation to dimethyl disulfide and dimethyl trisulfide. *Food Chem.* 49 387–392. 10.1016/0308-8146(94)90009-4

[B20] Dunleavy R. Lu L. Kiely C. J. McIntosh S. Berger B. W. (2016). Single-enzyme biomineralization of cadmium sulfide nanocrystals with controlled optical properties. *Proc. Natl. Acad. Sci. U.S.A.* 113 5275–5280. 10.1073/pnas.1523633113 27118834 PMC4868489

[B21] Durán-Toro V. Gran-scheuch A. Órdenes-aenishanslins N. Monrás J. P. Saona L. A. (2014). Quantum dot-based assay for Cu 2 + quantification in bacterial cell culture. *Anal. Biochem. J.* 450 30–36. 10.1016/j.ab.2014.01.001 24433980

[B22] Faraon A. Englund D. Fushman I. Stoltz N. Petroff P. (2007). Local quantum dot tuning on photonic crystal chips. *Appl. Phys. Lett.* 90:213110 10.1063/1.2742789

[B23] Gallardo C. Monrás J. P. Plaza D. O. Collao B. Saona L. A. Durán-Toro V. (2014). Low-temperature biosynthesis of fluorescent semiconductor nanoparticles (CdS) by oxidative stress resistant Antarctic bacteria. *J. Biotechnol.* 187 108–115. 10.1016/j.jbiotec.2014.07.017 25064158

[B24] Glatstein D. A. Bruna N. Gallardo-Benavente C. Bravo D. Carro Pérez M. E. Francisca F. M. (2018). Arsenic and cadmium bioremediation by antarctic bacteria capable of biosynthesizing CdS fluorescent nanoparticles. *J. Environ. Eng.* 144:04017107 10.1061/(ASCE)EE.1943-7870.0001293

[B25] Holmes J. D. Richardson D. J. Saed S. Evans-Gowing R. Russell D. A. Sodeau J. R. (1997). Cadmium-specif ic formation of metal sulfide Q-particles’. *Microbiology* 143 2521–2530. 10.1099/00221287-143-8-2521 9274006

[B26] Hoo C. M. Starostin N. West P. Mecartney M. L. (2008). A comparison of atomic force microscopy (AFM) and dynamic light scattering (DLS) methods to characterize nanoparticle size distributions. *J. Nanoparticle Res.* 10 89–96. 10.1007/s11051-008-9435-7

[B27] Horcas I. Fernández R. Gómez-Herrero J. Baro A. M. (2007). WSXM: a software for scanning probe microscopy and a tool for nanotechnology. *Rev. Sci. Instrum.* 78:013705. 10.1063/1.2432410 17503926

[B28] Jadhav P. Bhand G. R. Mohite K. C. Chaure N. B. (2017). CdS quantum dots synthesized by low-cost wet chemical technique. *AIP Conf. Proc.* 1832 1–3. 10.1063/1.4980379

[B29] Jang G. G. Jacobs C. B. Ivanov I. N. Joshi P. C. Meyer H. M. Kidder M. (2015). In situ capping for size control of monochalcogenide (ZnS, CdS and SnS) nanocrystals produced by anaerobic metal-reducing bacteria. *Nanotechnology* 26:325602. 10.1088/0957-4484/26/32/325602 26207018

[B30] Jo J. H. Singh P. Kim Y. J. Wang C. Mathiyalagan R. Jin C. G. (2016). *Pseudomonas* deceptionensis DC5-mediated synthesis of extracellular silver nanoparticles. *Artif. Cells Nanomed. Biotechnol.* 44 1576–1581. 10.3109/21691401.2015.1068792 26232081

[B31] Kale A. Bao Y. Zhou Z. Prevelige P. E. (2013). Directed self-assembly of CdS quantum dots on bacteriophage P22 coat protein templates. *Nanotechnology* 24:045603. 10.1088/0957-4484/24/4/045603 23296127

[B32] Kertesz M. A. (2000). Riding the sulfur cycle - metabolism of sulfonates and sulfate esters in Gram-negative bacteria. *FEMS Microbiol. Rev.* 24 135–175. 10.1111/j.1574-6976.2000.tb00537.x 10717312

[B33] Kertesz M. A. (2001). Bacterial transporters for sulfate and organosulfur compounds. *Res. Microbiol.* 152 279–290. 10.1016/s0923-2508(01)01199-8 11421275

[B34] Kertesz M. A. (2004). Metabolism of sulphur-containing organic compounds. pseudomonas, biosynth. *Macromol. Mol. Metab.* 3 323–357. 10.1007/978-1-4419-9088-4_12

[B35] Khachatryan G. Khachatryan K. Stobinski L. Tomasik P. Fiedorowicz M. Lin H. M. (2009). CdS and ZnS quantum dots embedded in hyaluronic acid films. *J. Alloys Compd.* 481 402–406. 10.1016/j.jallcom.2009.03.011

[B36] Korpi A. Järnberg J. Pasanen A. (2009). Microbial volatile organic compounds. *Crit. Rev. Toxicol.* 39 139–193. 10.1080/10408440802291497 19204852

[B37] Kredich N. M. (2008). Biosynthesis of cysteine. *EcoSal Plus* 3 1–30. 10.1128/ecosalplus.3.6.1.11 26443742

[B38] Kumar C. G. Mamidyala S. K. (2011). Extracellular synthesis of silver nanoparticles using culture supernatant of *Pseudomonas aeruginosa*. *Coll. Surfaces B Biointer.* 84 462–466. 10.1016/j.colsurfb.2011.01.042 21353501

[B39] Kuznetsova Y. V. Rempel A. A. (2015). Size and zeta potential of CdS nanoparticles in stable aqueous solution of EDTA and NaCl. *Inorg. Mater.* 51 215–219. 10.1134/S0020168515020119

[B40] Lina P.-C. Lina S. Paul C. W. Rajagopalan S. (2014). Techniques for physicochemical characterization of nanomaterials. *Biotechnol. Adv.* 31 711–726. 10.1038/mp.2011.182 24252561 PMC4024087

[B41] Liu S. Wang X. Pang S. Na W. Yan X. Su X. (2014). Fluorescence detection of adenosine-5 0 -triphosphate and alkaline phosphatase based on the generation of CdS quantum dots. *Anal. Chim. Acta* 827 103–110. 10.1016/j.aca.2014.04.027 24833001

[B42] Lomans B. P. Van Der Drift C. Pol A. Op den Camp H. J. (2002). Microbial cycling of volatile organic sulfur compounds. *C. Cell. Mol. Life Sci.* 59 575–588. 10.1007/s00018-002-8450-6 12022467 PMC11337448

[B43] Lu X. Fan C. He W. Deng J. Yin H. (2013). Sulfur-containing amino acid methionine as the precursor of volatile organic sulfur compounds in algea-induced black bloom. *J. Environ. Sci.* 25 33–43. 10.1016/s1001-0742(12)60019-9 23586297

[B44] Mal J. Nancharaiah Y. V. Van Hullebusch E. D. Lens P. N. L. (2016). Metal chalcogenide quantum dots: biotechnological synthesis and applications. *RSC Adv.* 6 41477–41495. 10.1039/c6ra08447h

[B45] Marusak K. E. Feng Y. Eben C. F. Payne S. T. Cao Y. You L. (2016). Cadmium sulphide quantum dots with tunable electronic properties by bacterial precipitation. *RSC Adv.* 6 76158–76166. 10.1039/c6ra13835g 28435671 PMC5397112

[B46] McHugh K. J. Jing L. Behrens A. M. Jayawardena S. Tang W. Gao M. (2018). Biocompatible semiconductor quantum dots as cancer imaging agents. *Adv. Mater.* 30 1–18. 10.1002/adma.201706356 29468747

[B47] Mi C. Wang Y. Zhang J. Huang H. Xu L. Wang S. (2011). Biosynthesis and characterization of CdS quantum dots in genetically engineered *Escherichia coli*. *J. Biotechnol.* 153 125–132. 10.1016/j.jbiotec.2011.03.014 21458508 PMC3102602

[B48] Monrás J. P. Díaz V. Bravo D. Montes R. A. Chasteen T. G. Osorio-Román I. O. (2012). Enhanced glutathione content allows the in vivo synthesis of fluorescent CdTe nanoparticles by *Escherichia coli*. *PLoS One* 7:e48657. 10.1371/journal.pone.0048657 23185270 PMC3504078

[B49] Muntaz Begum S. K. Ravindranadh K. Ravikumar R. V. S. S. N. Rao M. C. (2016). Spectroscopic studies on PVA capped ZnSe nanoparticles. Optoelectron. *Adv. Mater. Rapid Commun.* 10 889–892.

[B50] Muthalif M. P. A. Sunesh C. D. Choe Y. (2019). Enhanced light absorption and charge recombination control in quantum dot sensitized solar cells using tin doped cadmium sulfide quantum dots. *J. Coll. Interface Sci.* 534 291–300. 10.1016/j.jcis.2018.09.035 30237116

[B51] Nguyen N. H. Duong T. G. Hoang V. N. Pham N. T. Dao T. C. Pham T. N. (2015). Synthesis and application of quantum dots-based biosensor. *Adv. Nat. Sci. Nanosci. Nanotechnol.* 6:15015 10.1088/2043-6262/6/1/015015

[B52] Nozik A. J. Beard M. C. Luther J. M. Law M. Ellingson R. J. Johnson J. C. (2010). Semiconductor quantum dots and quantum dot arrays and applications of multiple exciton generation to third-generation photovoltaic solar cells. *Chem. Rev.* 110 6873–6890. 10.1021/cr900289f 20945911

[B53] Oliva-Arancibia B. Órdenes-Aenishanslins N. Bruna N. Ibarra P. S. Zacconi F. C. Pérez-Donoso J. M. (2017). Co-synthesis of medium-chain-length polyhydroxyalkanoates and CdS quantum dots nanoparticles in *Pseudomonas* putida KT2440. *J. Biotechnol.* 264 29–37. 10.1016/j.jbiotec.2017.10.013 29056529

[B54] Plaza D. O. Gallardo C. Straub Y. D. Bravo D. Pérez-Donoso J. M. (2016). Biological synthesis of fluorescent nanoparticles by cadmium and tellurite resistant Antarctic bacteria: exploring novel natural nanofactories. *Microb. Cell Fact.* 15 1–11. 10.1186/s12934-016-0477-8 27154202 PMC4858823

[B55] Qin Z. Yue Q. Liang Y. Zhang J. Zhou L. Hidalgo O. B. (2018). Extracellular biosynthesis of biocompatible cadmium sulfide quantum dots using trametes versicolor. *J. Biotechnol.* 284 52–56. 10.1016/j.jbiotec.2018.08.004 30107199

[B56] Rengers C. Nikolai G. Eychmüller A. (2019). “Quantum dots and quantum rods,” in *Biological Responses to Nanoscale Particles. NanoScienc*, eds Gehr P. Zellner R. (Cham: Springer).

[B57] Richards S. Baker M. A. Wilson M. D. Lohstroh A. Seller P. (2016). Femtosecond laser ablation of cadmium tungstate for scintillator arrays. *Opt. Lasers Eng.* 83 116–125. 10.1016/j.optlaseng.2016.03.004

[B58] Sambrook J. Russell D. W. (2001). *Molecular Cloning, A Laboratory Manual*, 3rd Edn Cold Spring: Cold Spring Harbor Laboratory Press.

[B59] Sanchez F. Sobolev K. (2010). Nanotechnology in concrete - a review. *Constr. Build. Mater.* 24 2060–2071. 10.1016/j.conbuildmat.2010.03.014

[B60] Sankhla A. Sharma R. Yadav R. S. Kashyap D. Kothari S. L. Kachhwaha S. (2016). Biosynthesis and characterization of cadmium sulfide nanoparticles - an emphasis of zeta potential behavior due to capping. *Mater. Chem. Phys.* 170 44–51. 10.1016/j.matchemphys.2015.12.017

[B61] Schulz S. Dickschat J. S. (2007). Bacterial volatiles: the smell of small organisms. *Nat. Prod. Rep.* 24 814–842. 10.1039/b507392h 17653361

[B62] Shatalin K. Shatalina E. Mironov A. Nudler E. (2011). H2S: a universal defense against antibiotics in bacteria. *Science* 334 986–990. 10.1126/science.1209855 22096201

[B63] Siegbahn K. Nordling C. Fahlman A. Nordberg R. Hamrin K. (1967). ESCA-atomic, molecular and solid state structure studied by means of electron spectroscopy Nova Acta Regiae Soc. *Sci. Upsaliensis Ser. IV* 20:84.

[B64] Solomon A. (2018). The emergence of nanotechnology and its applications. *Res. J. Nanosci. Eng.* 2 8–12.

[B65] Song Y. Huang M. Luo D. Zhong D. Hou H. (2014). Effect of CdS QDs linked functional groups on interaction between CdS QDs and EcoRI. *Coll. Surfaces A Physicochem. Eng. Asp.* 444 299–306. 10.1016/j.colsurfa.2013.12.074

[B66] Syed A. Ahmad A. (2013). Extracellular biosynthesis of CdTe quantum dots by the fungus *Fusarium oxysporum* and their anti-bacterial activity. *Spectrochim. Acta A Mol. Biomol. Spectrosc.* 106 41–47. 10.1016/j.saa.2013.01.002 23357677

[B67] Tandon S. Vats S. (2016). Microbial biosynthesis of cadmium sulfide (Cds) nanoparticles and their characterization. *Eur. J. Pharm. Med. Res.* 3 545–550. 12115128

[B68] Ulloa G. Collao B. Araneda M. Escobar B. Álvarez S. Bravo D. (2016). Use of acidophilic bacteria of the genus *Acidithiobacillus* to biosynthesize CdS fluorescent nanoparticles (quantum dots) with high tolerance to acidic pH. *Enzyme Microb. Technol.* 95 217–224. 10.1016/j.enzmictec.2016.09.005 27866618

[B69] Ulloa G. Quezada C. P. Araneda M. Escobar B. Fuentes E. álvarez S. A. (2018). Phosphate favors the biosynthesis of CdS quantum dots in *Acidithiobacillus thiooxidans* ATCC 19703 by improving metal uptake and tolerance. *Front. Microbiol.* 9:234. 10.3389/fmicb.2018.00234 29515535 PMC5826283

[B70] Venegas F. A. Saona L. A. Monrás J. P. Órdenes-Aenishanslins N. Giordana M. F. Ulloa G. (2017). Biological phosphorylated molecules participate in the biomimetic and biological synthesis of cadmium sulphide quantum dots by promoting H2S release from cellular thiols. *RSC Adv.* 7 40270–40278. 10.1039/c7ra03578k

[B71] Vermeij P. Kertesz M. A. (1999). Pathways of assimilative sulfur metabolism in *Pseudomonas* putida. *J. Bacteriol.* 181 5833–5837. 10482527 10.1128/jb.181.18.5833-5837.1999 PMC94106

[B72] Wagner A. M. Knipe J. M. Orive G. Peppas N. A. (2019). Quantum dots in biomedical applications. *Acta Biomater.* 94 44–63. 10.1016/j.actbio.2019.05.022 31082570 PMC6642839

[B73] Waly S. A. Shehata M. M. Mahmoud H. H. (2017). Synthesis and characterization of CdS nanoparticles prepared by precipitation in the presence of span 20 as surfactant. *Russ. J. Appl. Chem.* 90 292–297. 10.1134/S1070427217020203

[B74] Wang D. Li X. Zheng L. L. Qin L. M. Li S. Ye P. (2018). Size-controlled synthesis of CdS nanoparticles confined on covalent triazine-based frameworks for durable photocatalytic hydrogen evolution under visible light. *Nanoscale* 10 19509–19516. 10.1039/c8nr06691d 30320326

[B75] Wu R. Wang C. Shen J. Zhao F. (2015). A role for biosynthetic CdS quantum dots in extracellular electron transfer of *Saccharomyces cerevisiae*. *Process Biochem.* 50 2061–2065. 10.1016/j.procbio.2015.10.005

[B76] Yang Z. Lu L. Berard V. F. He Q. Kiely C. J. Berger B. W. (2015). Biomanufacturing of CdS quantum dots. *Green Chem.* 17 3775–3782. 10.1039/c5gc00194c 31122428

[B77] Yang Z. Lu L. Kiely C. J. Berger B. W. McIntosh S. (2016). Biomineralized CdS quantum dot nanocrystals: optimizing synthesis conditions and improving functional properties by surface modification. *Ind. Eng. Chem. Res.* 55 11235–11244. 10.1021/acs.iecr.6b03487

[B78] Zhou M. Ghosh I. (2007). Quantum dots and peptides: a bright future together. *N. Z. Med. J.* 88 325–339. 17167795 10.1002/bip.20655

