# Supplementary material for: Biosynthesis of CdS Quantum Dots Mediated by Volatile Sulfur Compounds Released by Antarctic Pseudomonas fragi
Source: Front Microbiol. 2019 Aug 13;10:1866. doi: 10.3389/fmicb.2019.01866 (PMC6700389; doi:10.3389/fmicb.2019.01866)
Supplement: Supplementary file 1 [file Table_1.DOCX]

Supplementary Material

## Supplementary Figures


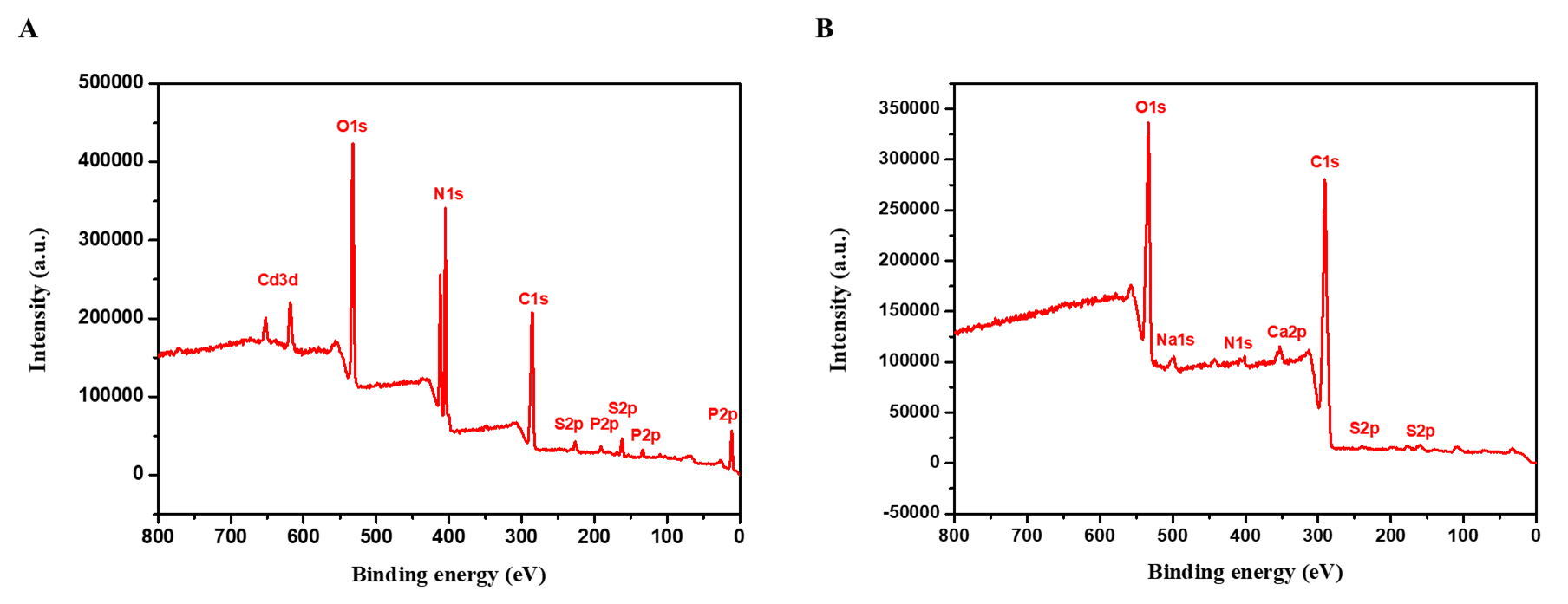


**Supplementary Figure S1.** XPS survey spectra of CdS nanoparticles obtained with (A) Cys, (B) Met.


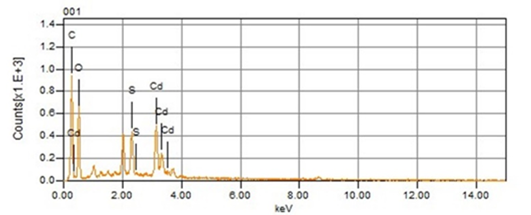


**Supplementary Figure S2**. EDX graph of CdS QDs biosynthesized by *P. fragi* GC01 with Cys for 3h (orange).


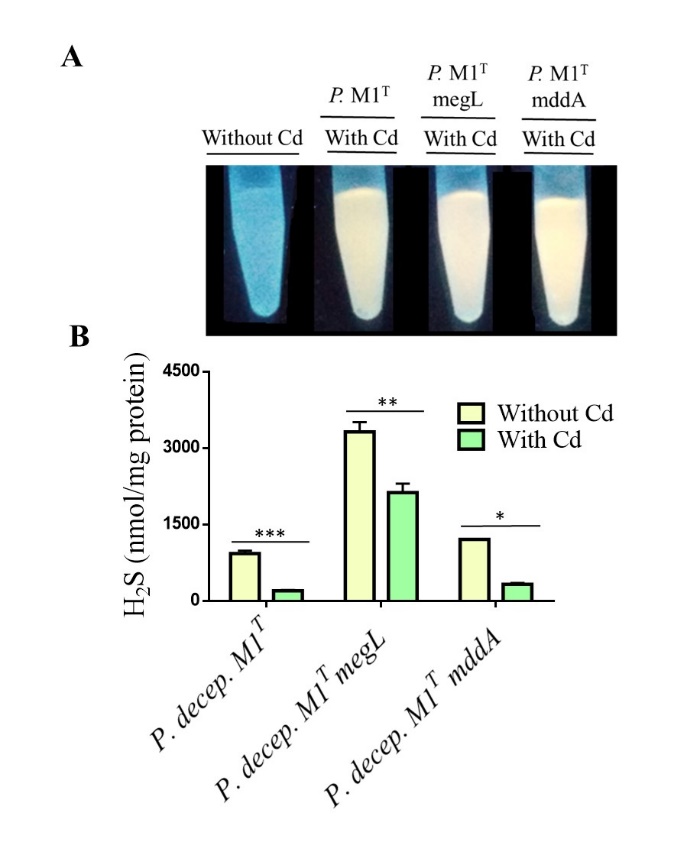


**Supplementary Figure S3.** VSCs produced by *Pseudomonas deceptionensis* M1^T^ strains under biosynthesis conditions with Cys. (**A**) Fluorescence of bacterial supernatants under biosynthesis conditions during 1 h after UV light exposure. (**B**) Quantiﬁcation of H_2_S by GC after 1 h incubation. Bacterial strains were grown under biosynthesis conditions in M9 medium with 2mM cysteine, in presence or absence of CdCl_2_ 20 µg mL at 28°C. Error bars represent standard deviation (n=3). Student’s t-test (P<0.05): Comparison between treatments without and with cadmium (Cd). *Statistically significant differences.
